# Addressing the unresolved challenge of quantifying skiing exposure—A proof of concept using smartphone sensors

**DOI:** 10.3389/fspor.2023.1157987

**Published:** 2023-05-09

**Authors:** Anita Meinke, Jörg Spörri, Luzius Brogli, Patrick Schwab, Walter Karlen

**Affiliations:** ^1^ Mobile Health Systems Lab, Institute of Robotics and Intelligent Systems, Department of Health Sciences and Technology, ETH Zurich, Zurich, Switzerland; ^2^ Sports Medical Research Group, Department of Orthopaedics, Balgrist University Hospital, University of Zurich, Zurich, Switzerland; ^3^ University Centre for Prevention and Sports Medicine, Department of Orthopaedics, Balgrist University Hospital, University of Zurich, Zurich, Switzerland; ^4^ Institute of Biomedical Engineering, University of Ulm, Ulm, Germany

**Keywords:** athlete, monitoring, mHealth, sensors, training diary, injury prevention, alpine skiing

## Abstract

In epidemiological studies related to winter sports, especially alpine skiing, an unresolved methodological challenge is the quantification of actual on-snow activity exposure. Such information would be relevant for reporting meaningful measures of injury incidence, which refers to the number of new injuries that occur in a given population and time period. Accordingly, accurate determination of the denominator, i.e., actual “activity exposure time”, is critical for injury surveillance and reporting. In this perspective article, we explore the question of whether wearable sensors in combination with mHealth applications are suitable tools to accurately quantify the periods in a ski day when the skier is physically skiing and not resting or using a mechanical means of transport. As a first proof of concept, we present exemplary data from a youth competitive alpine skier who wore his smartphone with embedded sensors on his body on several ski days during one winter season. We compared these data to self-reported estimates of ski exposure, as used in athletes' training diaries. In summary, quantifying on-snow activity exposure in alpine skiing using sensor data from smartphones is technically feasible. For example, the sensors could be used to track ski training sessions, estimate the actual time spent skiing, and even quantify the number of runs and turns made as long as the smartphone is worn. Such data could be very useful in determining actual exposure time in the context of injury surveillance and could prove valuable for effective stress management and injury prevention in athletes.

## Introduction

In winter sports such as alpine skiing, both competitive and recreational, the risk of injury is extraordinarily high (1, 2). With respect to the international Olympic committee (IOC) consensus recommendations on the methods for recording and reporting epidemiological data in sports (3), an unresolved methodological challenge in alpine skiing is the accurate quantification of actual activity exposure on snow, representing the denominator of the most commonly used measures of injury incidence (e.g., injuries/1,000 skiing hours). As such, recording activity exposure as time spent on the hill is too simplistic, since the actual exposure time is the time spent skiing down the slope, not the time spent on the chairlift or in the breaks between different runs. In fact, accurate tracking of phases of skiing down the slope and making turns or jumps is technically challenging, and to date, primarily self-reported estimates of ski exposure are used in athletes' training diaries.

One potential solution to this unresolved problem may be wearable sensors and mHealth applications. Sensors are commonly used to describe movements in various sports (4). Specifically, in alpine skiing, inertial measurement units (IMUs), global positioning systems (GPSs), pressure-sensitive insoles, and surface electromyography (sEMG) have been applied to analyse skiers' kinematics, kinetics, and muscle activation patterns (5–23). An overview of their application and the resulting methodological consequences for further use has recently been addressed in two review articles (24, 25). Furthermore, it has been suggested that sensor-based approaches may provide feedback to enhance sports performance or prevent injury in general, not just for competitive athletes (26).

The setups and systems described above have been employed to describe individual runs and skiing sessions. However, simpler systems might quantify the total exposure to skiing throughout an entire day of skiing or season. A recent review summarizing data across different sport disciplines suggested that increased training loads during certain time periods may promote injury and therefore encourage regular monitoring (27). More specifically for skiing, it has been reported that higher training intensity and volume are generally associated with an increased risk of illness in young athletes, although an association with injuries was not found (28). In addition, Fröhlich et al. (29) confirmed an association of training quantity with a more frequent occurrence of persistent complaints after the return of severe knee injuries in elite athletes. However, both studies rely on self-reported estimates of load exposure, and to the best of our knowledge, no study has yet employed sensor-based approaches in the context of alpine skiing. Nevertheless, self-reported estimates can only quantify actual exposure to a very limited and imprecise extent. This constraint could be overcome by using sensors and mHealth applications to accurately quantify skiing exposure.

In this perspective article, we explore the question of whether smartphone sensors combined with mobile applications could be a suitable option for quantifying actual on-snow activity exposure. For this purpose, we present exemplary data from a youth competitive alpine athlete who wore his smartphone (including sensors) on his body during several ski days in the same winter season and self-reported estimates of ski exposure commonly used in athletes' training diaries.

## Materials and methods

### Data collection

Out of the potential participant pool underlying the studies of Schoeb et al. (30, 31), one youth competitive alpine skier for whom a large amount of smartphone app-based data was available was selected for illustration of this perspective article. The skier carried a smartphone on his body during several ski days and self-reported estimates of ski exposure commonly used in athletes' training diaries into the app during a single winter season. The study was carried out according to the Declaration of Helsinki and authorized by the cantonal ethics committee Zurich (KEK-ZH-NR: 2018-01807), and the participant provided written informed consent.

### Smartphone app

A custom smartphone app was developed for research purposes within the Swiss National Research Program 75 “Big Data” (167302). The app was designed to record data from different embedded sensors, such as acceleration, speed, and altitude, based on the accelerometer and the GPS signals. Sensor data were only collected when the phone moved, and acceleration data were saved in sequences of 15 min to keep the size of the files manageable. In addition to recording data from the smartphone sensors, short self-report forms could be submitted. Recorded data were sent to a server when the phone was connected to Wi-Fi and stored in a MongoDB (MongoDB Inc., New York City, USA) database. The app design was compatible with preserving privacy while providing high-resolution data. The credentials for login of the participant were stored separately from the data. A connection between the data from the app and other relevant study data was made by the participant reporting a token to the investigators that could be accessed within the participant's installation of the app and communicated to the study staff. The participant was encouraged to log his ski training sessions by using the app. Each report included 4 questions, prompting the participant to indicate the date and the start time of his ski sessions. Furthermore, the time effectively spent skiing in hours and minutes and the number of runs made were surveyed.

### Data analysis

The data were processed and explored in MATLAB (MathWorks, Natick, USA) and R (32). The altitude and speed of the smartphone were derived from the database and segmented to contain data per day only. Records were removed that were shorter than 20 altitude samples after eliminating missing values or where more than 10 altitude samples had the value 0. In addition, we removed data which was entirely below 1700m and records for which the average speed was lower than 1 m/s and the moving mean of 1/15^th^ of the signal turned to zero during the record. These values have been chosen to achieve good cleaning results specifically in this data set. Duplicate records were discarded. Altitude was graphically displayed together with the acceleration data aggregated across axes. Each record received a rating into one of three categories: (1) the ski event and individual runs were visible; (2) skiing was visible but not individual runs; and (3) no skiing was visible on the record. Furthermore, for each record, the number of runs was counted, and the onset and end of each run were manually labelled. From these labels, the actual ski time was calculated for each ski event. Descriptive statistics and graphs, Pearson correlation, and chi-square tests as described by Field et al. (33) were used to describe the data obtained from the participant. The R package *gmodels* (34) was used for the chi-square test. The acceleration data were smoothed for graphical visualization.

## Results

### Manual data cleaning and data quality

Based on the sensor data, a total of 69 records were indicated as potentially containing ski events and were manually labelled. Three invalid duplicate entries were removed. Of the remaining 66 ski events, 11 were discarded from further analyses, as the ski event was not clearly visible during manual labelling. Examples of events for all three categories are displayed in Supplementary Figure S1. Of the remaining 55 events that were retained, 37 were labelled ski events but not individual runs, and 18 were labelled ski events and individual runs. Similar to Supplementary Figure S1C, some events indicated that the phone was left at a change room and not carried on the body during the training. Of the 66 self-reports, 4 were registered for dates that already had a report. One of these was verified as a date with two skiing sessions. Two of the duplicate reports were registered one month after the event, which may indicate that the participant mistakenly selected the wrong month. All self-reports were retained for further analysis. For all reports, the participant indicated the date for which the report was registered and the number of runs. In one report, the time of ski session start was missing, and 6 times the estimated skiing duration was not reported. One entry contained an unlikely estimated skiing duration of 796 min.

### Ski events identified using both data sources

A comparison of the ski events derived from self-reported data and manually labelled sensor data is shown in Supplementary Figure S2. During the entire period, skiing was registered for 71 unique dates based on both sources of data combined. One date contained two separate ski sessions, which were visible in the sensor data and registered by self-report. On 45 of the 71 dates, skiing was indicated by the self-report and the labelled sensor data. For 17 of the self-reported dates, no corresponding sensor data could be obtained, and for 9 of the dates with labelled sensor data, no skiing was reported. As indicated in Supplementary Figure S2, during the first month of study participation, most registered dates could also be detected in the sensor data. During the rest of the study, there were more days during which the self-reports and data derived from the sensors did not agree.

### Correlation between self-reported and sensor-based data

**Figure 1:**
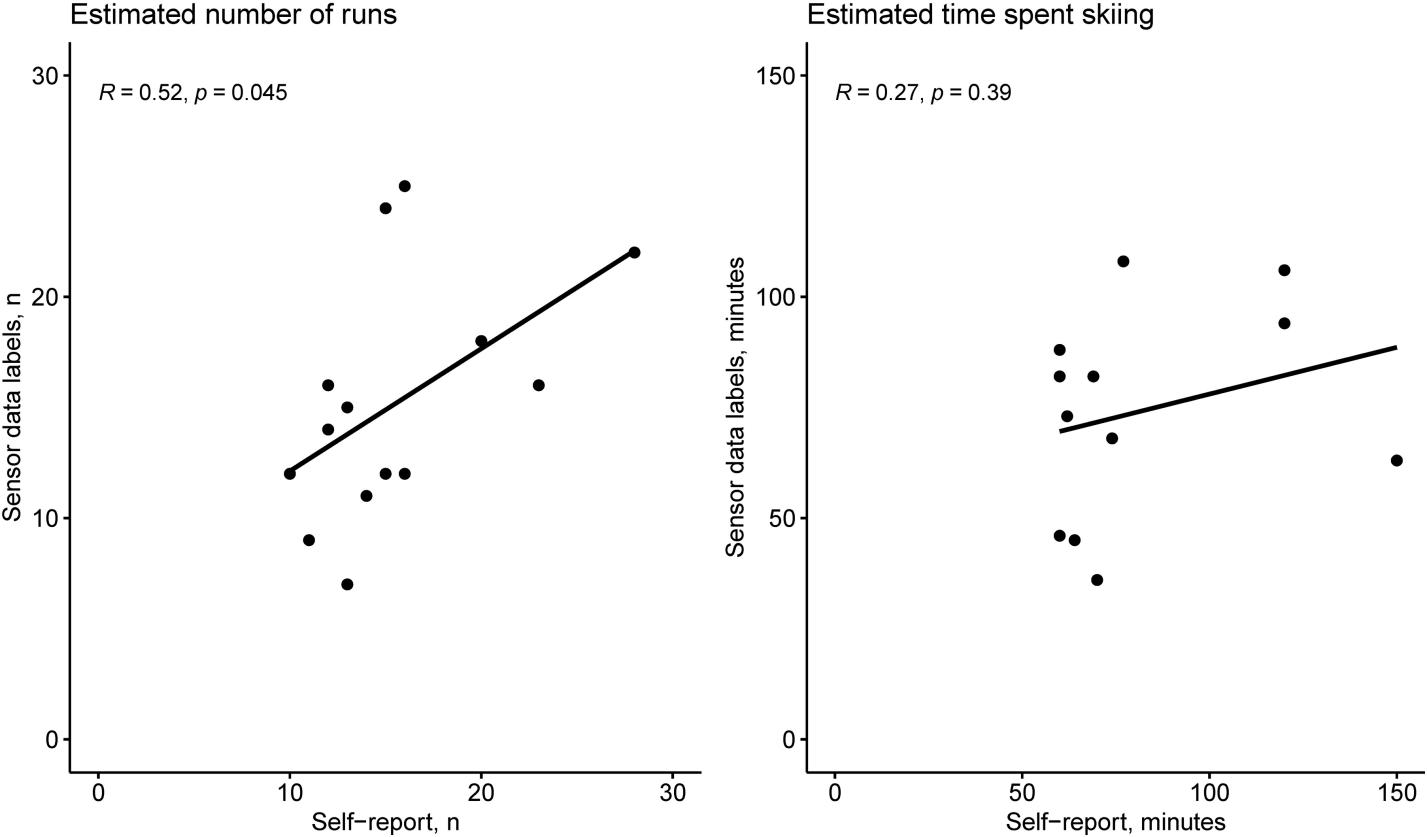
Scatter plots and Pearson correlation between self-reported and sensor-based data.

For those records that had complete estimates for the number of runs (*n *= 15) and for the time spent skiing (*n *= 12), correlations were calculated and are displayed in Figure 1. The graphs show that the number of runs moderately correlated between the self-reported data and the estimates based on the generated sensor data labels but not the estimated time spent skiing.

### Identification of runs and turns

**Figure 2:**
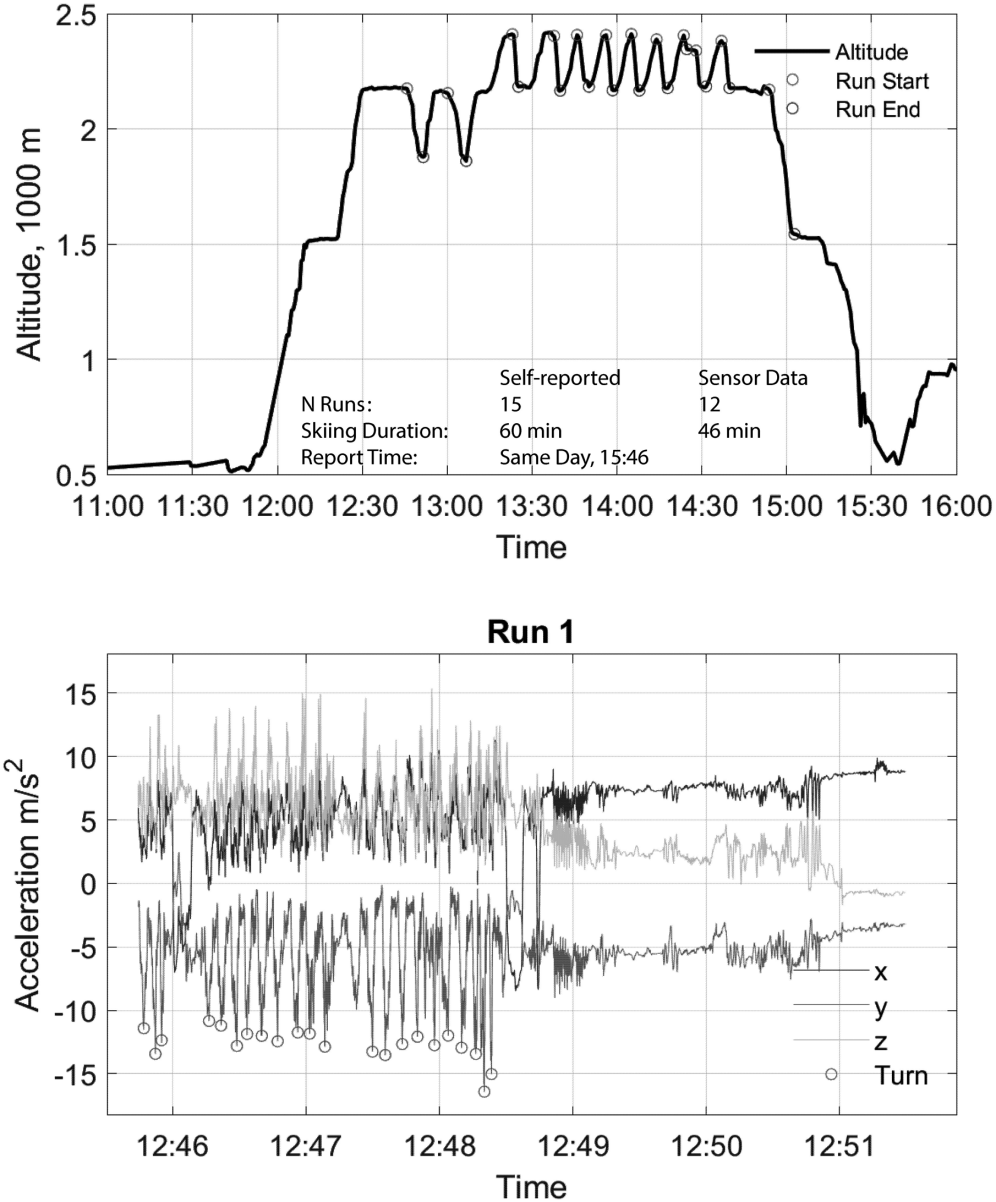
An ideal example of ski event detection based on altitude and acceleration and manual labels of run start and end for individual ski runs.

Data from high-quality records show that the number of runs and turns can be identified and characterized (Figure 2).

### Late reporting of ski events

Of all 66 self-reports, 39 were reported on the date of skiing and 27 at least 1 day later. The events reported later were registered at median during the following day. Except for two dates, where likely the wrong month had been selected, the delayed reports occurred at latest 5 days after the ski event. Supplementary Table S1 shows how many corresponding ski events were detected when the data were reported on the date of the event or later. Whether corresponding sensor data had been obtained was related to whether the ski event was manually registered for the current date or had been registered later *χ*^2^(1) = 6.89, *p *= 0.009. The odds ratio of 4.1 (CI: 1.23, 15.22) indicates that there was a higher chance of not finding corresponding sensor data when the self-report was submitted on a later date.

## Discussion

Based on the exemplary data presented in this perspective paper, we expect that it is technically feasible to quantify on-snow activity exposure in alpine skiing using data from sensors available in smartphones. Such an approach has several advantages over self-reported data, including but not limited to increased objectivity and accuracy. Thus, sensors could be well suited to record ski training sessions, estimate the time that was spent skiing and even quantify the number of performed runs and turns. These data may be helpful for incidence-based injury recording and reporting but may also have potential for effective load management in athletes. However, based on the exemplary data presented, three aspects are outstanding.

First, technical requirements would need to be satisfied more consistently. Sensor data collected from an uncontrolled setting are frequently less accurate than data collected in an experimental setting, and the identification of potential error sources is not trivial. As seen in the exemplary data, for several days, altitude and speed data derived from the GPS signal were missing and therefore rendered the available data incomplete. In a remote setting, the circumstances under which flawed data are obtained might be unknown, and developers of such systems might not have access to affected devices. Although many potential challenges could be circumvented by rigorous testing of the system and online algorithms could be used to detect difficulties early on, maintenance of the system will likely require continuous effort to ensure completeness and quality of such data. For example, updates of operating systems could make adaptation of such an app necessary.

Second, both self-report and sensor-based methods require active engagement of the athlete. Even though making use of the phones' built-in sensors seems perfectly effortless at first glance, minimal actions are still required from the user. The phone still must be brought to the training site and carried on the body throughout skiing. Based on the data we obtained, it was suspected that the phone might have been frequently left in a change room (e.g., Supplementary Figure S1C) instead of being worn on the body under the training suit. The actual ski session was not recorded in those cases, but time spent on the mountain was still captured. Using the phone must be proactively remembered right before the training, other than with self-reports where answers could be submitted offline at any time after the training. Especially at the beginning of the tracking period, for most self-reports, corresponding sensor data were available, which might be a sign for consistent reporting and use of the app. However, there were also events registered that had no corresponding self-report data, indicating that the sensors provided additional information regarding dates with training over the self-reported data. On the other hand, not all events that were included in the self-reported data were recorded by the sensors. To enhance the engagement of users, they could be provided with more detailed feedback in addition to the simple information about the number of reports submitted, which was the only available feedback in this study.

Third, provided that the first and second points are given, the suggested sensor-based approach can be considered adequate to provide detailed estimates of the number of ski events and duration of runs and to estimate the number of turns. As seen in the graphs showing details of records in good quality, these data appear suitable to quantify the actual ski time based on extracting individual runs, and furthermore, the number of turns performed during each run could be estimated. However, our exemplary data were collected for research purposes, and only minimal feedback was provided to the athlete to avoid biasing the results. When deploying such a system, more informative feedback could increase the engagement of athletes. In addition, as concluded by Düking et al. (35), care should be taken that the statistics offered to athletes actually add value to sport practice or prevention. For such applications, the objective should be made explicit, and the data should be protected, as athletes may have reservations that such information could adversely affect them (36).

A limitation of the presented data is that the definition of what constitutes a ski run was not clearly specified in advance and may have led to different interpretations by the athlete and investigators. Furthermore, manual labels were based on altitude alone. Although biases in self-reported data are well known, athletes may be able to accurately estimate the time spent skiing if they are able to recall the number of runs correctly. Run times have a high relevance for the sport and are frequently communicated; thus, self-reported estimates on the scale of individual runs could be of comparatively high quality.

To summarize our observations, phases of actual skiing were in general distinctly detectable based on the data collected from smartphone sensors, where data of sufficient quality were available. The development of a system for unobtrusive monitoring of on-snow activity appears possible based on such data, although in our exemplary data, not all reported ski events could be captured. An app that monitors ski exposure based on sensor data but possibly offers the complementary option to report manually may account for these shortcomings and be suitable for use by youth and adolescent athletes. In addition, information that is harder to obtain from self-reports, such as the number of turns performed, could enrich state-of-the-art analysis.

## Data Availability

The original contributions presented in the study are included in the article/**Supplementary Material**, further inquiries can be directed to the corresponding author.
