# Supplementary material for: Addressing the unresolved challenge of quantifying skiing exposure—A proof of concept using smartphone sensors
Source: Front Sports Act Living. 2023 May 9;5:1157987. doi: 10.3389/fspor.2023.1157987 (PMC10203200; doi:10.3389/fspor.2023.1157987)
Supplement: Supplementary file 1 [file Datasheet1.pdf]

Supplementary Material

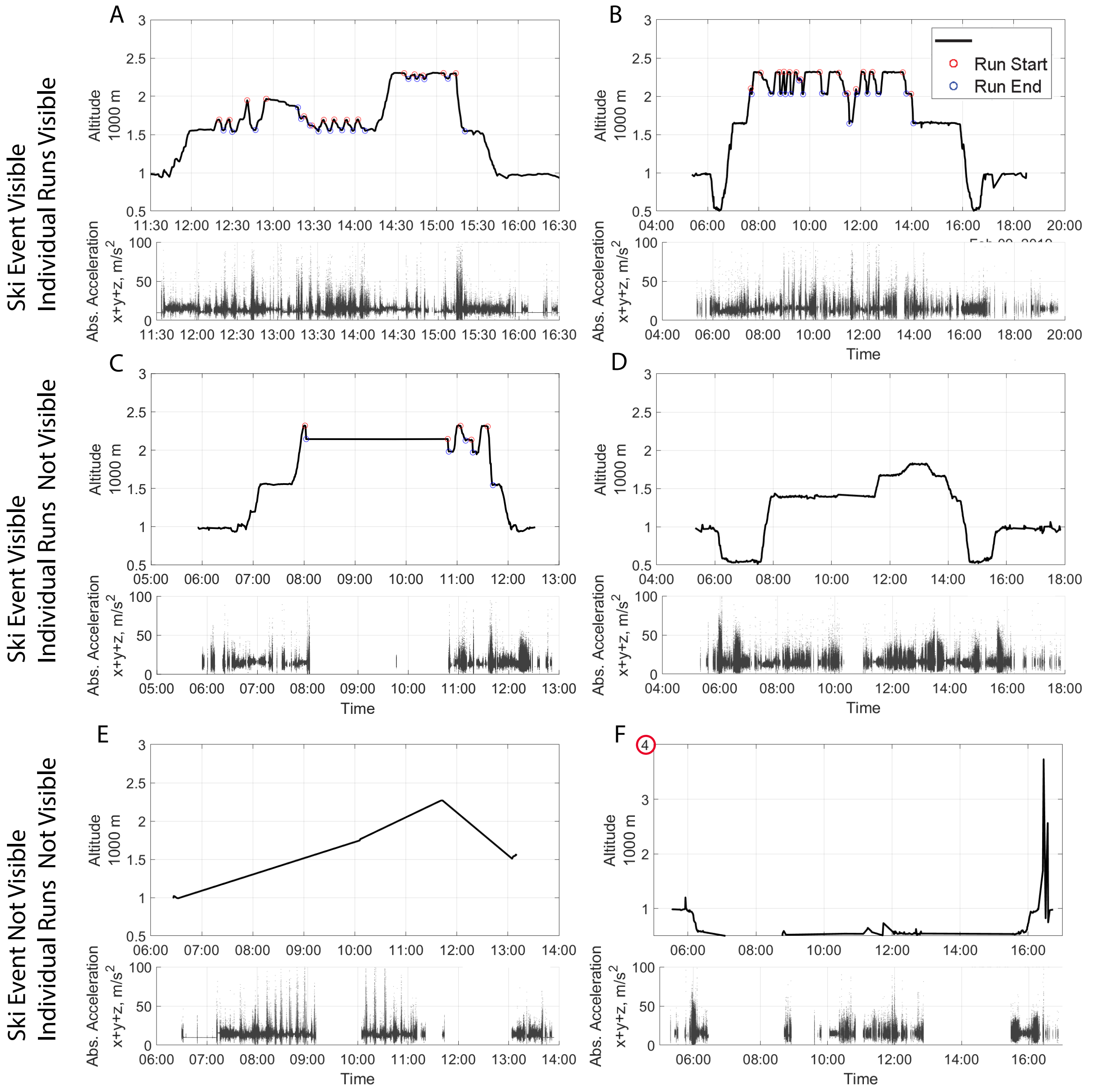

Figure S1. Altitude and acceleration for exemplary ski events with different visibility ratings. **(A)** and **(B)**: ski events and runs are visible. **(C)** and **(D)**: ski events are visible, and runs are not visible. **(E)** and **(F)**: ski events and runs are not visible.

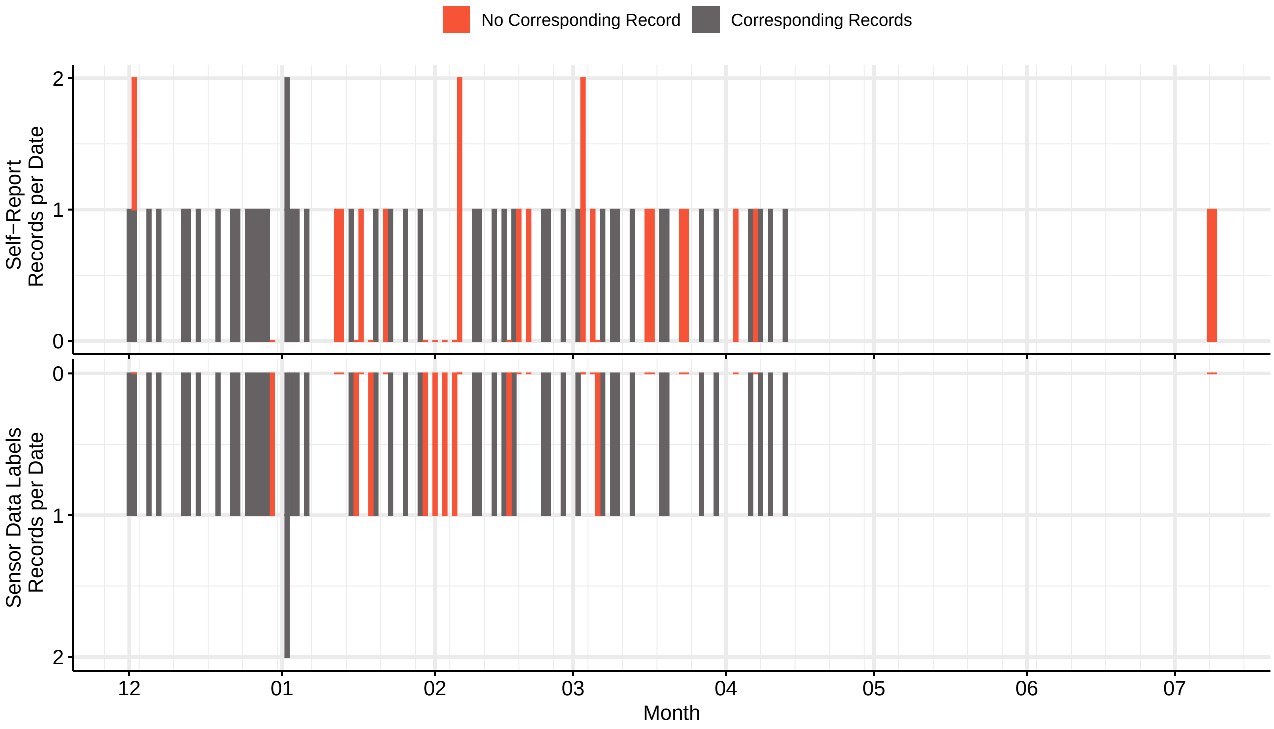

Figure S2. (A): Ski events detected based on sensor data. **(B)**: Ski events reported.
