# Supplementary material for: Addressing the unresolved challenge of quantifying skiing exposure—A proof of concept using smartphone sensors
Source: Front Sports Act Living. 2023 May 9;5:1157987. doi: 10.3389/fspor.2023.1157987 (PMC10203200; doi:10.3389/fspor.2023.1157987)
Supplement: Supplementary file 2 [file Table1.pdf]

Supplementary Table 1

Supplementary Table 1. Number of self-reports for which sensor data are available, depending on whether the self-report was registered on the same date as the ski event or later.

	**Sensor Data Availability**		
Report date	Not Available	Available	Row sum
Later report date than ski event	13	14	27
The same date as ski event	7	32	39
Total	20	46	66
